# Summary of the new guidelines for prevention of Infective Endocarditis: Implications for the developing countries

**DOI:** 10.4103/0974-2069.41058

**Published:** 2008

**Authors:** P. Bobhate, Robin J. Pinto

**Affiliations:** Glenmark Cardiac Centre, Mumbai, India

Infective endocarditis (IE) is an uncommon but a life-threatening infection affecting patients with structural heart defects. Despite advances in diagnosis, antimicrobial therapy, surgical intervention and management of complications, IE continues to be associated with a high morbidity and mortality. Recently, guidelines for prevention of IE have been revised by the American Heart Association (AHA).[1]

The reasons for this change in recommendations are as follows:

1. Although it is assumed that dental procedures may cause IE in patients who are predisposed to it and that antibiotic prophylaxis is effective in preventing it, actual scientific proof is lacking.
2. The absolute risk for IE from a dental procedure is exceedingly small. In fact, current evidence suggests that majority of cases of IE are caused by transient bacteremia associated with routine daily activities (such as brushing of teeth and chewing food) rather than dental procedures.
3. The ability of antibiotics to reduce bacteremia associated with dental procedures is controversial.
4. There is a potential risk of serious adverse reactions to antibiotics (anaphylaxis is estimated to occur in 15 - 25 individuals per 1 million patients who receive a dose of penicillin)
5. Frequent prophylactic antibiotic treatment may encourage emergence of antibiotic resistant strains of organisms causing IE thereby increasing morbidity and mortality.
6. Finally the earlier AHA guidelines on prevention of IE were very complicated making them difficult to be implemented.

The focus of prophylaxis has now shifted to prevention of dental caries which may reduce the incidence of bacteremia from daily activities and may be more important than prophylactic antibiotic for any dental procedure.

In another major departure from the previous guidelines the AHA no longer recommends prophylaxis based solely on an increased lifetime risk of IE. Thus patients with congenital heart defects such as ventricular septal defect, bicommissural aortic valve and valvar pulmonary stenosis, who have an increased lifetime risk of IE would not require prophylaxis according to the new guidelines. This is because the absolute lifetime risk of IE is still very small. Rather, prophylaxis should only be administered to patients with conditions associated with increased risk of adverse outcome from endocarditis. These are listed in Table 1

**Table 1:** Conditions associated with increased risk of complications due to infective endocarditis

1.	Prosthetic cardiac valve or prosthetic material used for cardiac valve repair
2.	Previous infective endocarditis
3.	Congenital heart disease
	Unrepaired cyanotic heart disease, including palliative shunts and conduits
	Completely repaired CHD with prosthetic material or device, whether by surgery
	or catheter intervention in first 6 months after the procedure
	Repaired CHD with residual defects at or adjacent to the site of repair which
	prevents endothelization at the site of repair.
4.	Cardiac transplantation recipients who develop cardiac valvulopathy

Antibiotic prophylaxis should be administered as a single dose before the procedure. If not administered before the procedure, the dosage may be given up to two hours after the procedure. However, the post procedure dose should be considered only when the patient does not receive the dose prior to the procedure. The presence of fever or other manifestations of systemic infection should alert the provider to the possibility of IE. In these circumstances it is important to obtain blood cultures and other relevant tests prior to administration of antibiotics.

Procedures for which antibiotic prophylaxis needs to administered:

1. Manipulation of gingival tissue or periapical region of teeth or perforation of oral mucosa
2. Established infection of gastrointestinal (GI) or genitourinary tract (GU) or for those who receive antibiotic to prevent wound infection or sepsis associated with GI or GU procedure.
3. Patients with enterococcal urinary infection or colonization
4. An invasive procedure of the respiratory tract that involves biopsy or incision of the respiratory mucosa.
5. Any procedure involving infected skin, skin suture or musculoskeletal tissue
6. Placement of prosthetic valves or intravascular or intracardiac material.

Recommended antibiotic protocols are as follows:

1. Antibiotic regimen for a dental procedure is summarized in Table 2

**Table 2:** Antibiotic regimen for a dental procedure

Situation	Agent	Regimen single dose 30 to 60 min before the procedure	
		
		Adults	Children
Oral	Ampicillin	2g	50mg/kg
Unable to take oral	Ampicillin Or	2g IM or IV	
Medication	Cefazolin or ceftriazone	1g IM or IV	50 mg/kg IM or IV
Allergic to penicillins or ampicillin - oral	Cefalexin Or	2g	50 mg/kg
	Clindamycin Or	600 mg	20 mg/kg
	azithromycin or clarithromycin	500 mg	15 mg/kg
Allergic to penicillins or ampicillin and unable to take oral medication	Cefazolin or ceftriazone	1g IM or IV	50 mg/kg IM or IV
	Or	600 mgIM or IV	20 mg/kg IM or IV
	Clindamycin
2. For those with established infection of GI or GU tract or for those who receive antibiotics to prevent wound infection or sepsis associated with GI or GU procedures, prophylaxis should include antibiotics active against enterococci such as penicillin, ampicillin, piperacillin or vancomycin. However in a significant change from the previous guidelines, antibiotic prophylaxis is not recommended for patients who undergo routine GI or GU procedures, including diagnostic esophago-gastroduodenostomy or colonoscopy
3. In patients with enterococcal urinary infection or colonization, it is important to eradicate enterococci from the urine prior to an elective cystocopy or manipulation of the urinary tract. If the procedure is not elective, antimicrobial regimen should contain drugs active against enterococci such as amoxicillin or ampicillin. If the patient cannot take orally, vancomycin should be used
4. For patients who undergo an invasive respiratory tract procedure the choice of antibiotic would depend on the organism known or suspected to cause the infection. If streptococcal viridans is suspected the regimen is similar to that for dental procedure; if staphylococcus aureus is suspected the regimen should contain antistaphyloccocal penicillin, cephalosporin or vancomycin. If the patient cannot tolerate penicillins or if MRSA is suspected vancomycin should be administered.
5. Procedures involving infected skin or musculoskeletal tissue should be covered with antibiotics active against staphylococci and β hemolytic streptococci such as penicllin or cephalosporin. Vancomycin or clindamycin should be administered if patient is not able to tolerate β lactams
6. During the placement of prosthetic valves or any other material in the heart or in the vascular system, choice of antibiotics should depend on the antibiotic susceptibility of each hospital

## General guidelines for dental hygiene

The most effective prevention of IE, requires good oral hygiene and prevention of dental caries for which, parents should be advised to take following measures.[2]

- Keep an infant's gums clean by using a gauze wipe or soft cloth
- Maintain oral hygiene- Brushing of teeth should commence as soon as they erupt (morning and evening) and flossing between the teeth once every day as soon as teeth contact one another.
- Diet—after the eruption of the first teeth, the parent should provide fruit juices (not to exceed 1 cup per day) during meals only. Carbonated beverages should be excluded from the child's diet. Infants should not be placed in bed with a bottle containing anything other than water. Ideally, infants should have their mouths cleansed with a damp cloth after feedings.
- Delay of colonization—mothers should be educated to prevent early colonization of dental flora in their infants by avoiding sharing of utensils (i.e, shared spoons, cleaning a dropped pacifier with their saliva etc.)
- Be aware of “nursing bottle mouth.” Do not put the child to bed for a nap or a night's sleep with a bottle of sweetened liquid in its mouth (e.g. milk, formula, or fruit juices). When the child is sleeping, a decrease in salivary flow allows the sugary liquid to remain in the child's mouth for a long time, causing tooth decay
- Note that some liquid medications contain from 30% to 50% sucrose; examples are those used for heart disease, seizures, or recurrent infections. These sugar-laden oral medications are most often given before nap time or bedtime when salivary flow is diminished and will not wash away the liquids. Give the doses of medication when the child is awake, and have the child rinse thoroughly or drink water immediately after a dose.

Whether recently published guidelines[1] can be applied in developing countries such as India where oral hygiene is relatively poor, will need validation by data. Although, more episodes of IE can be prevented by attention to oro-dental hygiene than by chemoprophylaxis, regular dental health screening in cardiac patients is not pursued avidly in the developing countries. A small study from India found an appallingly low (11%) awareness about IE prophylaxis and dental hygiene at the time of first physician contact.[3] Surprisingly, it continued to remain low during the follow-up (59%) despite the parents being on a supervised IE prophylaxis education program.[3] This fact has been once again highlighted in a survey published in this issue of the Journal.[4]

Hence, physicians should reinforce the importance of dental hygiene at every contact with susceptible children and their families. Till these practices translate into better oral hygiene in the pediatric population, it may be prudent to continue following the earlier guidelines in developing countries.
